# Ni(OH)_2_@Ni core-shell nanochains as low-cost high-rate performance electrode for energy storage applications

**DOI:** 10.1038/s41598-019-44285-1

**Published:** 2019-05-23

**Authors:** Mario Urso, Giacomo Torrisi, Simona Boninelli, Corrado Bongiorno, Francesco Priolo, Salvo Mirabella

**Affiliations:** 1 MATIS IMM-CNR and Dipartimento di Fisica e Astronomia “Ettore Majorana”, Università di Catania, via S. Sofia 64, 95123 Catania, Italy; 2 IMM-CNR, Z.I. VIII Strada 5, 95121 Catania, Italy

**Keywords:** Electronic properties and materials, Batteries, Synthesis and processing

## Abstract

Energy storage performances of Ni-based electrodes rely mainly on the peculiar nanomaterial design. In this work, a novel and low-cost approach to fabricate a promising core-shell battery-like electrode is presented. Ni(OH)_2_@Ni core-shell nanochains were obtained by an electrochemical oxidation of a 3D nanoporous Ni film grown by chemical bath deposition and thermal annealing. This innovative nanostructure demonstrated remarkable charge storage ability in terms of capacity (237 mAh g^−1^ at 1 A g^−1^) and rate capability (76% at 16 A g^−1^, 32% at 64 A g^−1^). The relationships between electrochemical properties and core-shell architecture were investigated and modelled. The high-conductivity Ni core provides low electrode resistance and excellent electron transport from Ni(OH)_2_ shell to the current collector, resulting in improved capacity and rate capability. The reported preparation method and unique electrochemical behaviour of Ni(OH)_2_@Ni core-shell nanochains show potential in many field, including hybrid supercapacitors, batteries, electrochemical (bio)sensing, gas sensing and photocatalysis.

## Introduction

The growing world energy demand, the finite supply of fossil fuels and the climate change due to detrimental gas emission have attracted a great attention of researchers in renewable energy resources and related energy storage technologies. A large variety of energy storage devices has been developed so far, including batteries and supercapacitors^1^. Batteries store energy through diffusion controlled redox reactions in bulk electrode material, leading to high energy density. However, the low power density of batteries hinders their use in applications where high power is required. On the other hand, supercapacitors bridge the gap between conventional capacitors and batteries, providing energy density higher than conventional capacitors and power density higher than batteries^2^. Recently, novel supercapacitor-battery hybrid systems, namely, hybrid supercapacitors, have received increasing interest since they combine the high power density of a supercapacitor-like material (negative electrode) with the high energy density of a battery-like material (positive electrode)^3^.

Among the more investigated positive electrodes for hybrid supercapacitors are NiO and Ni(OH)_2_ owing to their low-cost, well-defined redox reactions, environmental friendliness, and high theoretical capacity (359 and 289 mAh g^−1^, respectively)^4^. It is worth noting that these materials exhibit the typical electrochemical features of batteries, and then the most significant feature is the specific capacity [mAh g^−1^]^5,6^. Nonetheless, they are often described in terms of specific capacitance [F g^−1^] which doesn’t allow a straightforward comparison in literature^4^.

The more efficient strategy to obtain high-capacity NiO and Ni(OH)_2_ electrode is to fabricate nanostructured materials. In fact, it has been demonstrated that NiO and Ni(OH)_2_-based nanostructures possess superior electrochemical properties due to their high surface to volume ratio, efficient electrolyte penetration and low resistance^7^. Over the past years, 0D (nanoparticles^8^), 1D (nanowires^9^), 2D (nanosheets^10^) and 3D (flower-like structure^11^, nanowalls^12^) nanostructures have been developed. Among them, 3D materials are the more advantageous ones, since their better connectivity results in higher electrical conductivity and improved mechanical stability^13^. In particular, 3D Ni(OH)_2_ nanowalls is considered one of the best electrode since it adds the advantage of a unique open nanoporous structure formed by a tight network of nanosheets (20 nm thick, 0.1 ÷ 2 μm height) with excellent flexibility^12^. Also, Ni(OH)_2_ nanowalls can be prepared by a simple, low-cost, low-temperature and large-area chemical bath deposition process (CBD)^7^. Despite these promising features, only a few nanostructures have a capacity close to the theoretical one^4^. Moreover, most of them suffer from poor rate capability, since capacity dramatically reduces at the high charge-discharge rates required for high power applications. This is commonly attributed to the poor electrical conductivities of NiO and Ni(OH)_2_-based materials^13^. An effective approach to improve the rate capability of NiO and Ni(OH)_2_ nanostructures is to deposit them onto highly conductive current collectors, such as Ni nanotubes arrays^14^, graphene nanosheets^15^, carbon nanotubes^16^, and carbon coated 3D copper structure^17^. Another favourable strategy is based on core-shell nanostructures which take advantage of the synergistic properties offered by the two components (electrochemically active shell, and high-conductivity core). Semiconductive (3D TiO_2_ nanowires arrays^18^) and metallic (3D Ni nanoparticles^19^ and Ni nanotubes arrays^20^) cores have been reported. However, a simple and cost-effective approach to fabricate core-shell nanostructured electrodes with high capacity and rate capability is still absent, limiting their transfer to commercial products^4,13^. Moreover, the effects of the conductive core on the charge storage process have not been fully clarified yet. Therefore, a detailed comprehension of the electrochemical behaviour of core-shell structures could lead to an effective improvement of the energy storage performances of these materials.

In this work, we present a simple and low-cost approach to fabricate a novel Ni(OH)_2_@Ni core-shell nanochains with superior specific capacity and rate capability. The relationships between electrochemical properties and core-shell structure are investigated and modelled. The innovative design of Ni(OH)_2_@Ni core-shell nanochains has potential applications in hybrid supercapacitors, lithium-ion batteries, electrochemical (bio)sensing, gas sensing and photocatalysis.

## Methods

### Synthesis

Ni foam substrates (1 × 1.5 cm^2^, Goodfellow, thickness 1.6 mm, porosity 95%, 20 pores cm^−1^) were rinsed with acetone, isopropanol and deionized water (MilliQ, 18 MΩ cm), and dried under N_2_ gas flow. Ni-based nanostructures were grown on cleaned substrates by chemical bath deposition (CBD). Solution for CBD was prepared by mixing 0.42 M NiSO_4_·6H_2_O (Alfa Aesar, 98%), 0.07 M K_2_S_2_O_8_ (Alfa Aesar, 97%) and 3.5 wt% ammonia (Merck, 30–33 wt% NH_3_ in H_2_O). The solution was heated up to 50 °C and kept at this temperature through a bain-marie configuration^21^. Substrates were immersed (1 × 1 cm^2^ area) in the solution for 20 min. Then, the samples were rinsed with deionized water to clean deposited film from unwanted microparticulate and dried in N_2_ gas. Some samples were further annealed at 350 °C for 60 min in Ar followed by 60 min in forming gas (FG, Ar:H_2_ 95:5 mixture). Finally, an electrochemical oxidation of the annealed films surface was performed by cyclic voltammetry (CV) (100 cycles in the potential range −0.2 ÷ 0.8 V at 50 mV s^−1^ scan rate).

Film mass after each synthesis step was measured with a Mettler Toledo MX5 Microbalance (sensitivity: 0.001 mg). Before weighing, samples were washed several times with deionized water, dried in N_2_ gas and put in an oven at 60 °C for 1 h.

### Characterization

The surface morphology of samples was characterized by using a Scanning Electron Microscope (Gemini field emission SEM Carl Zeiss SUPRA 25) while the structural and chemical properties of samples were investigated by Transmission Electron Microscope (JEOL JEM-2010F TEM) operating at 200 keV accelerating voltage. Samples for TEM observation were prepared by standard TEM specimen preparation techniques by using a flat Ni substrate.

Electrochemical oxidation process and measurements were performed at room temperature by using a potentiostat (VersaSTAT 4, Princeton Applied Research, USA) and a three-electrode setup with a platinum counter electrode, a saturated calomel electrode (SCE) as reference, Ni-based nanostructures as working electrodes (1 × 1 cm^2^ immersed area), in 1 M KOH (Sigma Aldrich, ≥85%) supporting electrolyte. CV curves were recorded at different scan rates (1 to 50 mV s^−1^) in the potential range −0.2 ÷ 0.8 V. Galvanostatic charge-discharge (GCD) tests were conducted at different current densities (1 to 64 A g^−1^) in the potential range 0 ÷ 0.4 V. Electrochemical impedance spectroscopy (EIS) was performed at 0 V vs open circuit potential with a superimposed 5 mV sinusoidal voltage in the frequency range 10^4^–10^−2^ Hz.

## Results

### Synthesis and characterization of Ni(OH)_2_@Ni core-shell nanochains

**Figure 1:**
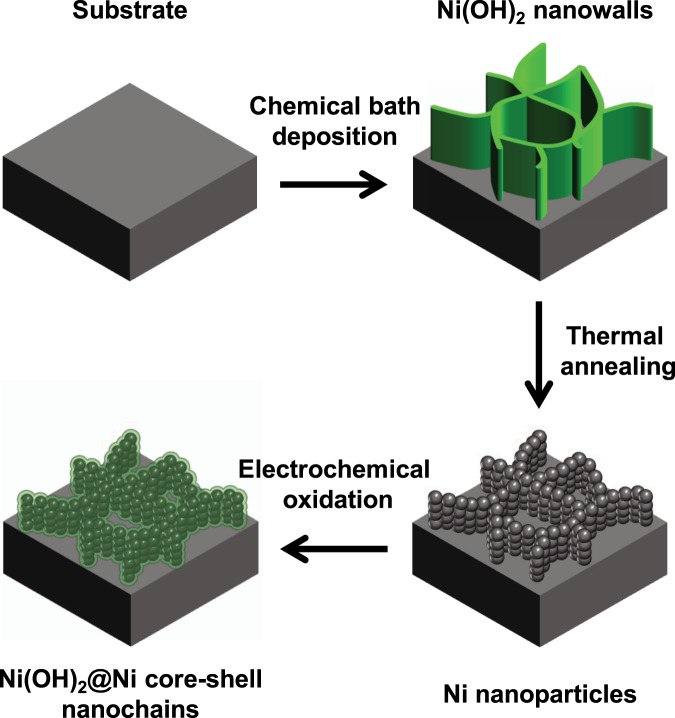
Schematic illustration of the three-step synthesis of Ni(OH)_2_@Ni core-shell nanochains.

**Figure 2:**
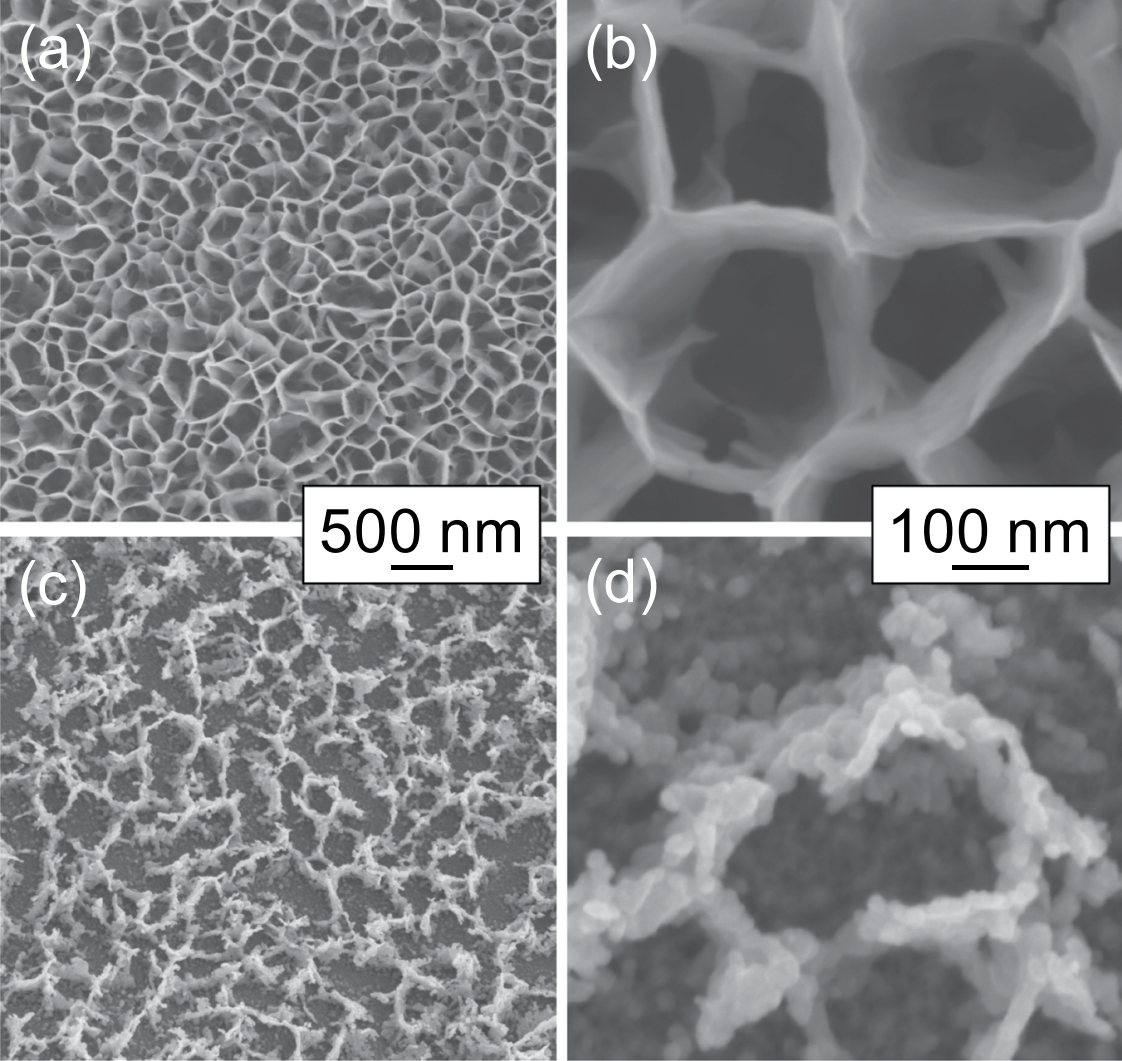
SEM images of (**a**), (**b**) Ni(OH)_2_ nanowalls and (**c**), (**d**) Ni nanoparticles at different magnifications.

A conductive core is required to enable faster electron transfer, and thus high-rate performance electrode. In this work a Ni(OH)_2_@Ni core-shell nanochains was obtained by a three-step synthesis shown in Fig. 1. The 50 °C CBD leads to a Ni(OH)_2_ nanowalls structure with thin (~10 nm) sheets mostly perpendicular to the substrate. Figure 2(a,b) report the SEM images at different magnifications of the film grown by CBD, which shows the typical morphological features of Ni(OH)_2_ nanowalls.

A reducing thermal process leads to a structural and chemical transformation. As shown in Fig. 2(c,d), Ni(OH)_2_ nanowall shaped film was transformed into chain-like clusters of metallic Ni nanoparticles (20–30 nm in size). XRD patterns before and after annealing confirmed the Ni(OH)_2_ → Ni transformation^22^.

**Figure 3:**
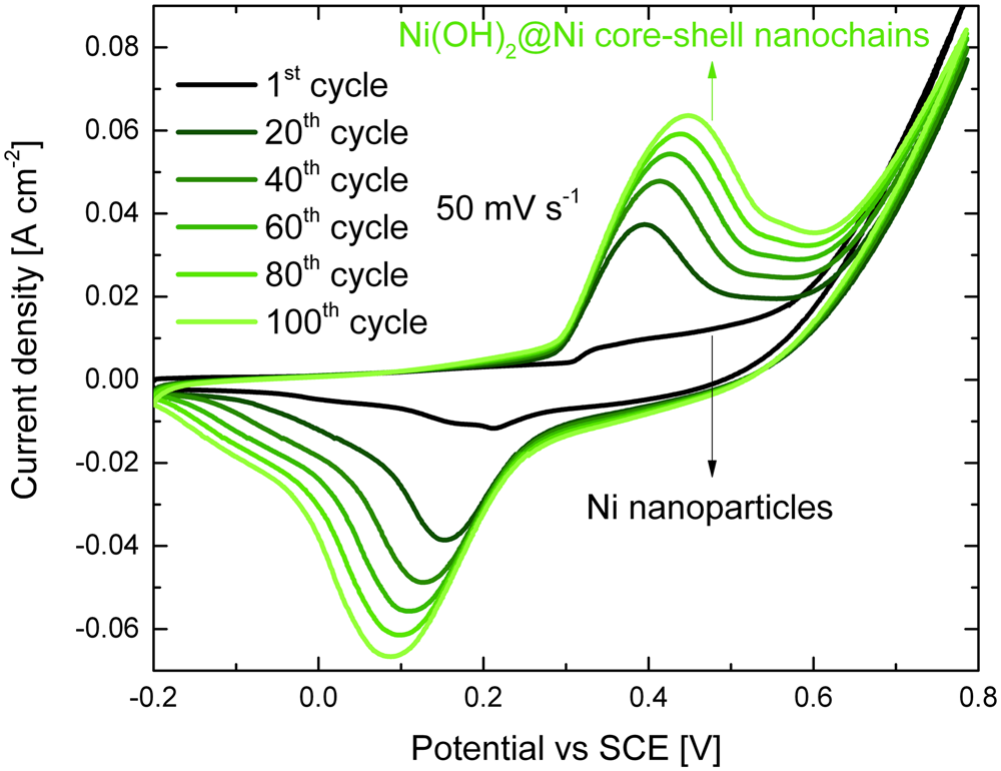
Electrochemical oxidation of the Ni nanoparticles performed by 100 CV cycles at 50 mV s^−1^ in the potential range −0.2 ÷ 0.8 V in 1 M KOH solution.

An electrochemical process is finally used to obtain the core-shell structure. Figure 3 reports the CV curves recorded during the electrochemical oxidation of the Ni nanoparticles. Two pronounced oxidation and reduction peaks appeared with increasing cycle number, which are attributed to the redox couple Ni^2+^/Ni^3+^. In fact, first Ni(OH)_2_ is formed because of the reaction between Ni nanoparticles surface and OH^−^ ions in solution^23^:

1 $${{\rm{Ni}}}^{{\rm{0}}}+2{{\rm{OH}}}^{-}\to {\rm{Ni}}{({\rm{OH}})}_{2}+2{{\rm{e}}}^{-}.$$

Then, the following reversible redox reaction occurs:

2 $${\rm{Ni}}{({\rm{OH}})}_{2}\leftrightarrow {\rm{NiOOH}}+{{\rm{e}}}^{-}+{{\rm{H}}}^{+}.$$

Peaks area enlargement with cycling is due to the increasing Ni(OH)_2_/NiOOH volume. The electrochemical oxidation was stopped after 100 CV cycles since almost stable curves were obtained (Fig. S1).

**Figure 4:**
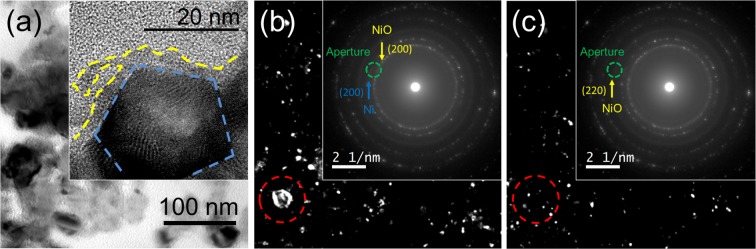
TEM images of Ni(OH)_2_@Ni core-shell nanochains: (**a**) Bright field (HR-TEM in inset), (**b**) and (**c**) dark field images for different position of objective aperture (green dashed circle) in the SAED pattern (inset).

Transmission electron microscopy analyses were performed to investigate the crystallinity of Ni(OH)_2_@Ni core-shell nanochains. Figure 4(a) reports a bright field image of the sample, displaying some bunches of nanoparticles with diameters ranging between 20–30 nm (a low-magnification image which shows the chain-like structure of the sample is reported in Fig. S2). The high resolution TEM (HR-TEM) image in the inset clearly demonstrates the core-shell structure, showing a 20 nm large nanoparticle surrounded by a 3–4 nm thin shell. The core presents a set of family planes with fringes separated by a distance equal to 1.8 Å while the shell displays two different family planes whose interplanar distance is equal to 2.1 and 2.3 Å. Such interplanar distances are compatible with the {200} Ni (1.8 Å), with the {200} NiO (2.1 Å) and the {101} Ni(OH)_2_ (2.3 Å), respectively. Selected Area Electron Diffraction ring-like patterns, acquired from the same region, are shown in the insets in Figs 4(b,c) and denote the polycrystalline nature of the sample. These results confirm the presence of the {200} Ni planes and the {200} and {220} NiO planes^14^. To better distinguish Ni from NiO domains, dark field images were acquired by putting the TEM objective aperture in correspondence of both the {200} Ni and {200} NiO diffracting rings (Fig. 4(b)) and in correspondence of the {220} NiO ring (Fig. 4(c)) in the SAED pattern. All images shown in Fig. 4 were acquired form the same region of TEM sample. It should be underlined that even by employing the smallest TEM objective aperture it was not possible to separate ring-like patterns corresponding to {200} Ni and {200} NiO, whose distance in the SAED is smaller than the objective aperture diameter (see the inset in Fig. 4(b)). Both large (~20 nm) and small (~3–4 nm) crystalline grains show high contrast in Fig. 4 (b) while Fig. 4(c) put in evidence only the presence of the small ones. In particular the large particle underlined in Fig. 4(b) is not visible in Fig. 4(c) where it appears surrounded by small nanocrystals. Moreover, the size of the large grains is consistent with that of the Ni nanoparticles, while the size of the small ones is comparable with the thickness of the NiO/Ni(OH)_2_ shell. In conclusion, this dark field visibility behaviour allows us strongly support that these core-shell nanostructures are formed by Ni crystalline grains surrounded by a ~3–4 nm NiO/Ni(OH)_2_ shell.

**Figure 5:**
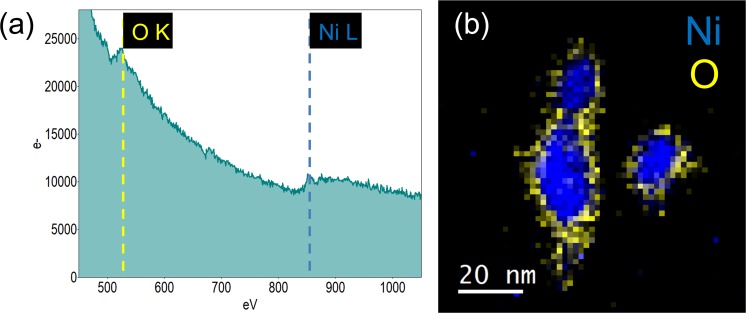
(**a**) Total EELS spectrum and (**b**) Elemental mapping of some core-shell nanoparticles.

Finally, it worth to be noted that both high resolution and dark field techniques are sensitive to the crystallography of nanomaterials thus, to strongly support the conclusions drawn so far, a chemical analysis at the nanoscale was conducted by means of STEM-Electron Energy Loss Spectroscopy (STEM-EELS) applied to about a ten of nanoparticles. First of all, the high energy EELS spectrum of each nanoparticle was acquired; as expected it shows the edge at 532 eV corresponding to the O_K_ ionization shell and the edge at 855 eV corresponding to the Ni_L_ shell (Fig. 5(a)). Secondly, the elemental mapping of Ni and O was generated on the base of this spectrum (Fig. 5(b)). It clearly demonstrates that the core shell structure is composed by a Ni core (in blue colour) surrounded by a uniform NiO shell (in yellow), in strong agreement with the HR-TEM investigation.

According to mass measurements (more details in the Supplementary Information), it was estimated that 36% Ni of Ni nanoparticles was consumed to form the Ni(OH)_2_ shell. The remaining Ni atoms (64%) constitute the highly conductive 3D backbone.

### Electrochemical properties of core-shell and nanowalls electrodes

**Figure 6:**
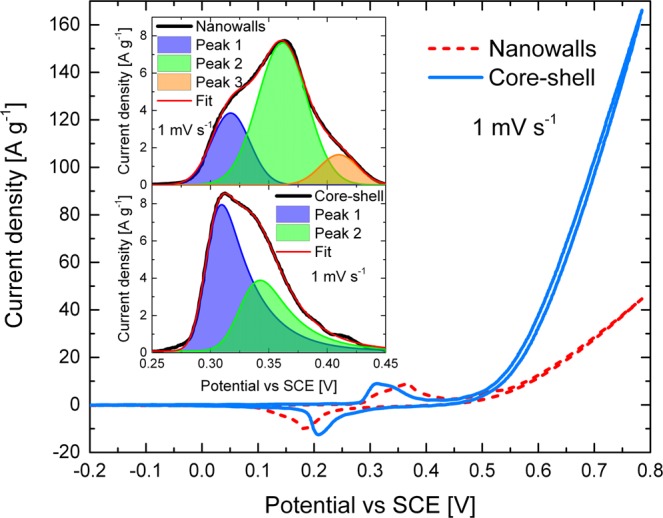
(**a**) Comparison of the CV curves of nanowalls (red dashed line) and core-shell (blue solid line) measured at 1 mV s^−1^ scan rate in the potential range −0.2 ÷ 0.8 V in 1 M KOH. The inset shows the fitted oxidation peaks of nanowalls (top) and core-shell (bottom).

CV was employed to identify the storage mechanism of Ni(OH)_2_ nanowalls (“nanowalls”) and Ni(OH)_2_ core-shell nanochains (“core-shell”) electrodes. Figure 6 compares the CV curves of the two samples at 1 mV s^−1^ scan rate in 1 M KOH. Both curves are very distinct from the classic rectangular shape of EDLCs and pseudocapacitors, showing instead the characteristic faradaic redox peaks of battery-like materials^4–6^.

The inset in Fig. 6 reports an enlarged scale of the oxidation peaks, revealing clear differences among the two electrodes. The peak of nanowalls (top inset) was fitted by a three-component model: peak 1 at ~0.320 V, peak 2 at ~0.360 V and peak 3 at ~0.410 V. Instead, the oxidation peak of core-shell (bottom inset in Fig. 6) was fitted by a two-component model: peak 1 at ~0.310 V, and peak 2 at ~0.340 V. Typically, CV peaks of Ni-based electrodes are associated to the redox reactions α-Ni(OH)_2_ ↔ γ-NiOOH and β-Ni(OH)_2_ ↔ β-NiOOH^24,25^. α-Ni(OH)_2_ is oxidized to γ-NiOOH at a lower potential than β-Ni(OH)_2_ oxidized to β-NiOOH^26^. Therefore, it can be reasonably concluded that peak 1 is related to γ-NiOOH formation, while peak 2 and 3 are related to β-NiOOH formation. Consequently, the reduction peak of nanowalls (~0.180 V) and core-shell (~0.210 V) is attributed to the totally overlapped components of α-Ni(OH)_2_ and β-Ni(OH)_2_ formation.

CV curves were also recorded at higher scan rates (Fig. S3). The shape of core-shell CV does not change significantly with increasing scan rate. This suggests a lower equivalent series resistance (ESR), which is the combined resistance of electrolyte and internal resistance of the sample^17^. As the scan rate increases, the oxidation and reduction peaks shift toward more positive and negative values, respectively. However, core-shell always presents a smaller separation among oxidation and reduction peaks than nanowalls, which is commonly associated to a better redox reversibility^24^.

**Figure 7:**
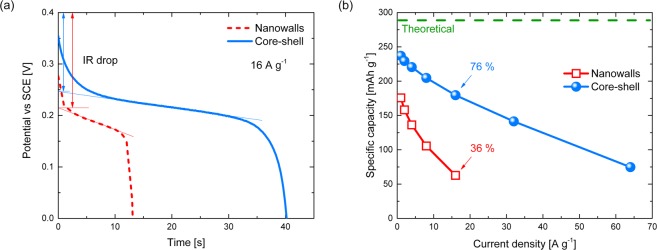
(**a**) Comparison of the discharge curves of nanowalls (red dashed line) and core-shell (blue solid line) measured at 16 A g^−1^ current density in 1 M KOH. (**b**) Specific capacity of nanowalls (red open squares) and core-shell (blue spheres) as function of current density.

The specific capacity (electrode capacity/mass of the active material, [mAh g^−1^]) is the more informative property to describe and compare the energy storage ability of different materials. Therefore, accurate mass measurements are required^4^. In this work particular care has been taken in evaluating the mass, and the obtained results are reported in Table S1. GCD tests at different current densities (Fig. S4) were performed to evaluate the specific capacity of the nanowalls and core-shell electrodes. Figure 7(a) compares the discharge profiles of the two samples at 16 A g^−1^. A voltage plateau is present in both curves, confirming the battery-like behaviour resulted from CV^4–6^. The voltage drop (IR drop) at the beginning of the discharge curves results from the ESR, which is the main contribution to energy and power loss at high charge-discharge rate. Core-shell clearly shows a lower IR drop, and thus a smaller ESR in agreement with CV measurements.

The specific capacity *Q*_*s*_ [mAh g^−1^] of the samples was calculated by^4^

3 $${{Q}}_{{s}}=\frac{{I}{\rm{\Delta }}{t}}{{m}\,}$$

where *I* is the constant current density [A cm^−2^], Δ*t* is the discharge time [s] and *m* is the mass of the active material [g cm^−2^]. Figure 7(b) reports the specific capacity as function of the current density for the two electrodes. The specific capacity decreases with increasing current density. However, the nanowalls electrode shows a specific capacity of 176 mAh g^−1^ at 1 A g^−1^ and 63 mAh g^−1^ at 16 A g^−1^, retaining 36%. Instead, the core-shell electrode shows a specific capacity of 237 mAh g^−1^ at 1 A g^−1^ and 180 mAh g^−1^ at 16 A g^−1^, retaining 76%. The superior rate capability of core-shell enabled even higher current densities. In particular, at the high current density of 64 A g^−1^ the specific capacity of core shell was still higher than that of nanowalls at 16 A g^−1^.

Specific capacity was also calculated from CV measurements (Fig. S5). The obtained results are consistent with those of GCD tests, indicating that core-shell has a superior charge storage ability, especially when high charge-discharge rates are considered.

The electrochemical utilization $z$ [%] of the active material can be calculated from GCD tests according to the equation^27^

4 $$z={Q}_{s}\,\frac{{M}_{Ni{(OH)}_{2}}}{F}\,100$$

where *Q*_*s*_ is the specific capacity [C g^−1^], ${M}_{Ni{(OH)}_{2}}$ is the molar mass of Ni(OH)_2_ (92.7 g mol^−1^) and *F* is the Faraday constant (96485 C mol^−1^). $z={\rm{100}} \%$ means that the whole active material undergoes redox reactions. The $z$ values of the two samples at different current densities are reported in Table 1. At 1 A g^−1^ 61% Ni(OH)_2_ in nanowalls and 82% in core-shell are used respectively. Such a difference is even more pronounced at higher current densities, as expected from Fig. 7(b). In fact, at 16 A g^−1^ only 22% Ni(OH)_2_ in nanowalls is used, while 62% in core-shell is still involved in the redox process. This result suggests an improved electrochemical utilization of the active material in core-shell.

**Table 1:** Electrochemical utilization of the active material z [%] in nanowalls and core-shell according to Equation (4).

Current density [A g^−1^]	z [%]	
	Nanowalls	Core-shell
1	61	82
2	55	79
4	47	76
8	37	71
16	22	62
32	/	49
64	/	26

**Figure 8:**
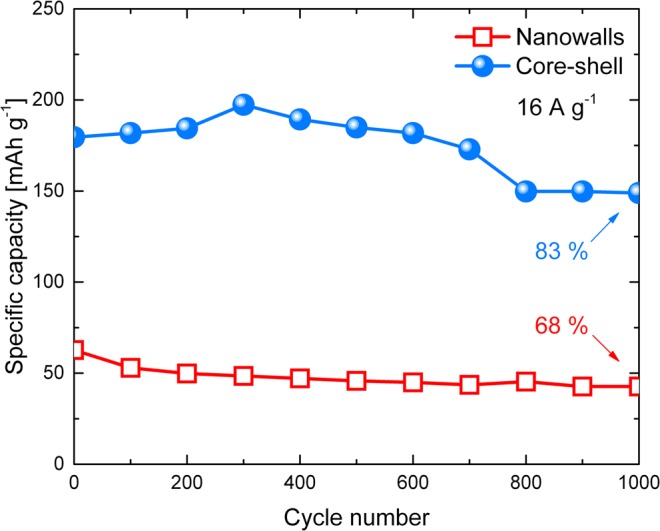
Cycling performances of nanowalls (red open squares) and core-shell (blue spheres) for 1000 GCD cycles at 16 A g^−1^ current density in 1 M KOH.

Ni-based electrodes suffer from significant capacity decay during charge-discharge cycles because redox reactions are involved^2^. Since a long cycling stability is critical for industrial applications, a stability test was performed by 1000 GCD cycles at the high current density of 16 A g^−1^. Figure 8 compares the cycling characteristics of the nanowalls and core-shell electrodes. After 1000 cycles the nanowalls electrode presents a specific capacity of 43 mAh g^−1^, retaining 68% of the initial value. The capacity decay can be explained by the growth of crystals size, leading to decrease in surface area, and to Ni(OH)_2_ flaking off caused by the volume change during charge-discharge as confirmed by SEM analysis after cycling tests (Fig. S6)^26^. Instead, the core-shell electrode shows first a rise in specific capacity (1–300 cycles) attributed to the fully activation of the Ni cores, followed by a decay (300–800 cycles), and finally a nearly constant capacity (800–1000 cycles). The capacity decay from cycle 300 to 1000 can’t be ascribed to morphological variations as demonstrated by SEM analysis after cycling tests (Fig. S6). To explain this behaviour it should be noted that α-Ni(OH)_2_ ↔ γ-NiOOH contributes more than β-Ni(OH)_2_ ↔ β-NiOOH to the core-shell energy storage process (inset in Fig. 6). However, α-Ni(OH)_2_ is unstable in water and typically recrystallizes into β-Ni(OH)_2_ with cycling^25,28^. Moreover, β-Ni(OH)_2_ ↔ β-NiOOH has a lower theoretical capacity than α-Ni(OH)_2_ ↔ γ-NiOOH^26,29^. Therefore, it can be reasonably concluded that the α-Ni(OH)_2_ → β-Ni(OH)_2_ transformation is responsible for the core-shell capacity decay. The complete transformation into β-Ni(OH)_2_ after 800 cycles determines a nearly constant capacity of 149 mhA g^−1^ (83% of the initial value). This value is higher than specific capacitance of nanowalls after 1000 cycles, indicating a better cycling stability of the core-shell electrode.

## Discussion

**Figure 9:**
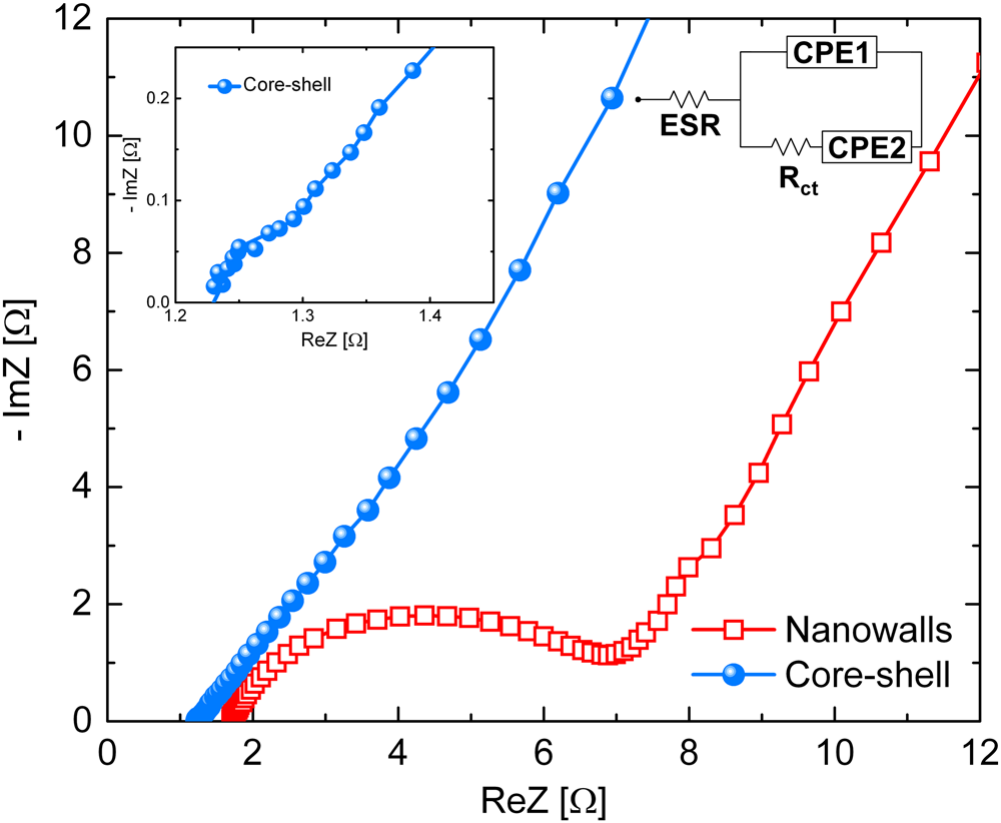
Nyquist plot of the nanowalls (red open squares) and core-shell (blue spheres) samples recorded at 0 V vs open circuit potential with a 5 mV superimposed AC voltage in the frequency range 10^4^ ÷ 10^−2^ Hz in 1 M KOH solution (the inset is the magnified high-frequency region of the core-shell electrode). Equivalent circuit model for the Nyquist plots is also reported: an equivalent series resistance (ESR) is connected in series with a constant phase element (CPE1) in parallel with the charge transfer resistance (Rct) and a constant phase element (CPE2)^14^.

To consolidate the improved energy storage properties of the core-shell sample, electrochemical impedance spectroscopy (EIS) analyses were performed. Figure 9 compares the Nyquist plots of nanowalls and core-shell obtained from EIS experiments, with an enlarged scale for core-shell at high frequencies (inset). The two plots show a semicircle arc in the high-frequency region, and a straight line in the low-frequency region. This shape can be modelled by the equivalent circuit reported in Fig. 9. The slopes of the two lines at low frequencies are similar, indicating a low ions diffusion resistance and a behaviour close to that of an ideal capacitor (line parallel to the imaginary axis). The ESR can be evaluated as the intercept on the real axis of the Nyquist plot. The lower ESR of core-shell (~1.2 Ω) than nanowalls (~1.7 Ω) is due to the presence of Ni cores, which leads a smaller IR drop in the discharge curves for fixed current density (Fig. 7(a)). The charge transfer resistance (R_ct_) can be measured as the diameter of the semicircle in the high-frequency region. It can be seen that core-shell has a lower R_ct_ (~0.1 Ω) than nanowalls (~5.4 Ω).

**Figure 10:**
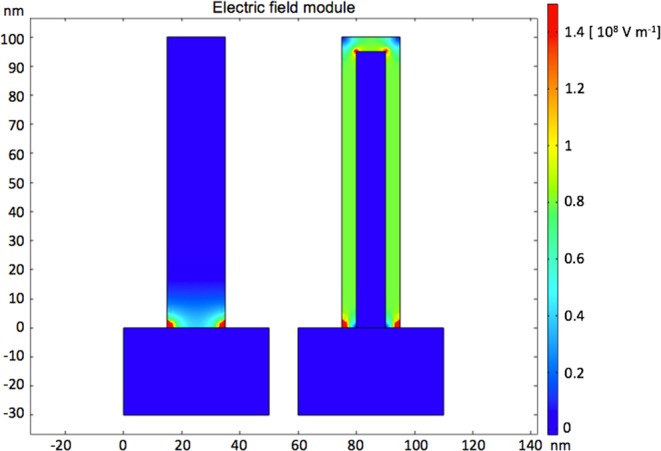
Electric field module distribution of the nanowalls (left) and core-shell (right) electrodes in 1 M KOH solution for 0.4 V applied bias, as simulated by COMSOL Multiphysics software. A 20 nm thick Ni(OH)_2_ nanosheet was used to simulate the nanowalls electrode. A 17 nm thick Ni nanosheet surrounded by a 3 nm thick Ni(OH)_2_ shell was used to simulate the core-shell electrode. The following room temperature conductivities were used: 10^−13^ S cm^−1^ for Ni(OH)_2_, 10^5^ S cm^−1^ for Ni, and 0.2 S cm^−1^ for 1 M KOH^28,36^.

To further support the excellent electrochemical behaviour of the core-shell, the electric field in the nanowalls and core-shell electrodes was simulated by using the COMSOL Multiphysics software (more details in the Supplementary Information). The resulted electric field module distributions are presented in Fig. 10 in false colour scale from 0 to 1.5 × 10^8^ V m^−1^. Nanowalls (left), displays a moderate electric field (~0.4 × 10^8^ V m^−1^) near the nanosheet/substrate interface, which is dramatically reduced going far away from the substrate. Instead, the conductive Ni core in core-shell (right) provides a uniform and enhanced electric field (~0.8 × 10^8^ V m^−1^) along the entire shell, in agreement with the improved electrochemical utilization reported in Table 1.

**Figure 11:**
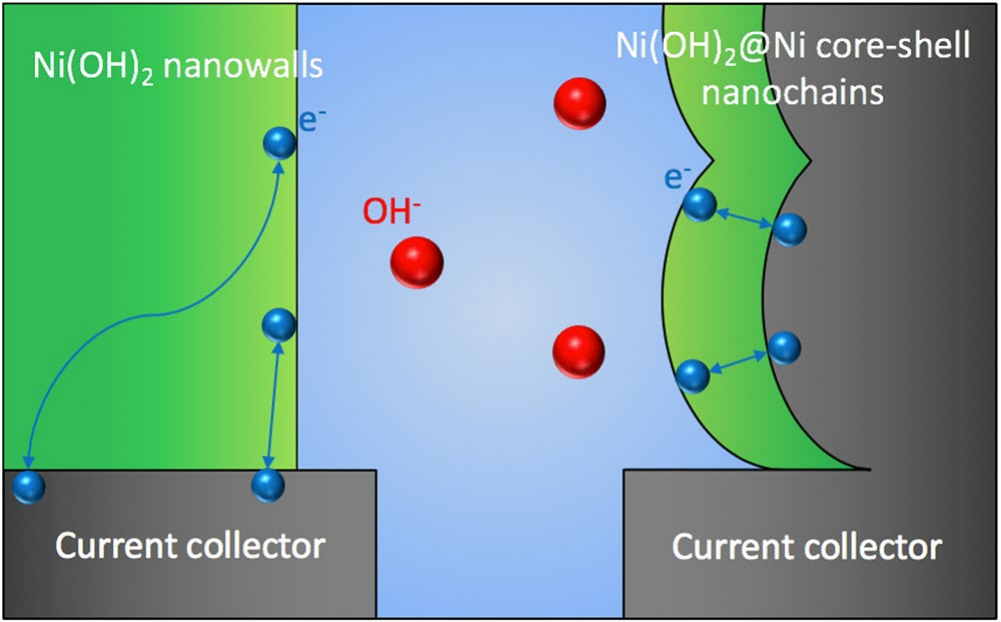
Schematic illustration of the electronic transport in the nanowalls (left) and core-shell (right) electrodes.

On the basis of the aforementioned results, the following model was developed to explain the improved specific capacity, rate capability and cycling stability of Ni(OH)@Ni core-shell nanochains. i) Ni cores reduce electrode resistance (thus energy dissipation) and charge-transfer resistance. Moreover, they enhance the electric field in the whole Ni(OH)_2_ shell. These features result in higher charge storage ability and faster redox process. ii) the 3 nm thin Ni(OH)_2_ shell shortens the electrons migration paths from the surface of the active material to the current collector (schematic illustration in Fig. 11). Also, a high OH^−^ ions diffusion coefficient of (1.415 ± 0.002) × 10^−6^ cm^2^ s^−1^ was estimated for the core-shell electrode according to the Randles-Sevcik equation (more details in the Supplementary Information), which is higher than 2.491 × 10^–7^ cm^2^ s^−1^ reported for Ni(OH)_2_/graphene nanosheets. Thanks to these unique features high charge/discharge rates can be supported. iii) the *in situ* electrochemical oxidation process allows a good contact between Ni(OH)_2_ shell and Ni cores. As a consequence, Ni(OH)_2_ can easily relax volume change during charge/discharge cycles, showing a good stability.

Table 2 compares the energy storage performances of recent Ni-based nanostructured electrodes. Excellent capacity retention at high charge-discharge rate is required for the development of commercial high power devices. From Table 2 it can be seen that even at high charge-discharge current density our Ni(OH)_2_@Ni core-shell nanochains shows higher specific capacity than most of the previous reports^11,15,30–33^. A few reports present comparable specific capacity values, however they were recorded at lower current density^18,34^. Su *et al*. reported a similar Ni(OH)_2_@Ni core-shell obtained electrode with a higher specific capacity but lower rate capability^19^, while Jiang and co-workers obtained a specific capacity higher than Ni(OH)_2_ theoretical limit by using a NiMoO_4_@Ni(OH)_2_ core-shell electrode where both core and shell are active materials^35^. The good cycling stability of the Ni(OH)_2_@Ni core-shell nanochains can be further improved by using graphene nanosheets^15^ or carbon nanotubes^31^.

**Table 2:** Energy storage performances of recent Ni-based nanostructured electrode (specific capacity [mAh g^−1^] values with “~” were calculated from specific capacitance [F g^−1^] reported in the relative ref.).

Year	Electrode material	Method	Specific capacity [mAh g^−1^]	Current density [A g^−1^]	Cycling stability		Ref.
					Cycle number	Retention [%]	
2014	Au NP-deposited Ni(OH)_2_	Hydrothermal + colloidal deposition	~142	20	5000	80	^30^
2014	Ni(OH)_2_ nanosheets on Ni foam	Wet environment + solution oxidation	~179	5	3000	75	^34^
2014	Amorphous Ni(OH)_2_@3D core-shell nanostructures	Electrodeposition	~269	20	3000	30	^19^
2015	Ni@NiO core-shell nanoparticles tube arrays	Electrodeposition + oxidation	~110	25	1000	92	^20^
2015	3D TiO_2_@Ni(OH)_2_ core-shell arrays	Hydrothermal + annealing + CBD	178	10	/	/	^18^
2015	CNT@Ni(OH)_2_ core-shell composites	CBD	~53	20	1000	92	^31^
2015	NiMoO_4_@Ni(OH)_2_ core/shell nanorods	Hydrothermal + electrodeposition	~328	16	1000	72	^35^
2016	3D flower-like β-Ni(OH)_2_	Solvothermal	~37	10	600	90	^11^
2016	Ni(OH)_2_/graphene nanosheets	Carbonization + hydrothermal	~156	10	1000	97	^15^
2017	Ni-Co double hydroxide	Electrodeposition	~140	20	5000	96	^32^
2018	Ni(OH)_2_ nanosheets on hollow mesoporous carbon spheres	Solvothermal + precipitation	~154	10	3000	81	^33^
2018	Ni(OH)_2_ nanowalls	CBD	63	16	1000	68	This work
2018	Ni(OH)_2_@Ni core-shell nanochains	CBD + annealing + CV	180	16	1000	83	This work

## Conclusions

In conclusion, we reported a novel Ni(OH)@Ni core-shell nanochains with promising high-rate energy storage performances. The core-shell structure consists of a 3D nanostructured Ni core embedded in a thin Ni(OH)_2_ shell. The Ni core is formed by interconnected chains-like clusters of Ni nanoparticles (20–30 nm), grown by a low-cost CBD of Ni(OH)_2_ nanowalls and a low-temperature annealing process. The Ni(OH)_2_ shell is made of nanocrystalline grains (3–4 nm), obtained by the *in situ* electrochemical oxidation of the Ni film surface. The electrochemical behaviour of the sample is dominated by faradaic redox processes (battery-like signature), which enabled a high capacity of 237 mAh g^−1^ at 1 A g^−1^, a high rate capability (76% at 16 A g^−1^, 32% at 64 A g^−1^), and good stability (83% capacity retention after 1000 cycles at 16 A g^−1^). These remarkable features if compared with those of similar NiO and Ni(OH)_2_-based nanostructures are attributed to the high surface area, faster electron transport, enhanced electric field and improved utilization of the active material provided by the core-shell design. As a result, Ni(OH)_2_@Ni core-shell nanochains has potential in many applications, including hybrid supercapacitors, batteries, electrochemical (bio)sensing, gas sensing and photocatalysis. Finally, the reported low-cost preparation of core-shell nanostructures can be used to improve the electrochemical performances of other existing NiO and Ni(OH)_2_-based electrodes.

## Supplementary information

Supplementary Information
