# Supplementary material for: Ni(OH)2@Ni core-shell nanochains as low-cost high-rate performance electrode for energy storage applications
Source: Sci Rep. 2019 May 23;9:7736. doi: 10.1038/s41598-019-44285-1 (PMC6533347; doi:10.1038/s41598-019-44285-1)
Supplement: Supplementary file 1 — Supplementary Information [file 41598_2019_44285_MOESM1_ESM.pdf]

$Ni(OH)_2$@Ni core-shell nanochains as low-cost high-rate performance electrode for energy storage applications

Mario Urso[1*], Giacomo Torrisi[1], Simona Boninelli[2], Corrado Bongiorno[2], Francesco Priolo[1] and Salvo Mirabella[1]

[1] MATIS IMM-CNR and Dipartimento di Fisica e Astronomia "Ettore Majorana", Università di Catania, via S. Sofia 64, 95123 Catania, Italy.

[2] IMM-CNR, Z.I. VIII Strada 5, 95121 Catania, Italy.

E-mail: mario.urso@ct.infn.it

Supplementary information

$Ni(OH)_2$@Ni core-shell nanochains was obtained by the electrochemical oxidation of the Ni nanoparticles. Electrochemical oxidation was performed at room temperature by 100 CV cycles at 50 mV s^{-1} scan rate in the potential range -0.2÷0.8 V in 1 M KOH by using a three-electrode setup (Pt as counter electrode, SCE as reference electrode, 1x1 cm^2 Ni nanoparticles as working electrode).

The area of a CV curve represents the total charge Q [C cm^{-2}] exchanged in the cycle. The charge was calculated every 10 CV cycles (charge at the n*10th cycle $Q_{n*10}, n = 1, \ldots, 10$). Then, $\Delta Q/Q$ [%] was calculated as

$$\frac{\Delta Q}{Q} = \frac{Q_{n*10} - Q_{(n-1)*10}}{Q_{(n-1)*10}} * 100 \qquad n = 1, \ldots, 10$$

where Q_0 $(n = 1)$ is the charge of the 1th CV cycle. Figure S1 shows $\frac{\Delta Q}{Q}$ as function of the cycle number. $\frac{\Delta Q}{Q}$ increases from cycle 1 to 20, then decreases. Finally, $\frac{\Delta Q}{Q}$ is almost constant (cycles 70-100), suggesting a total electrochemical oxidation of the Ni nanoparticles.

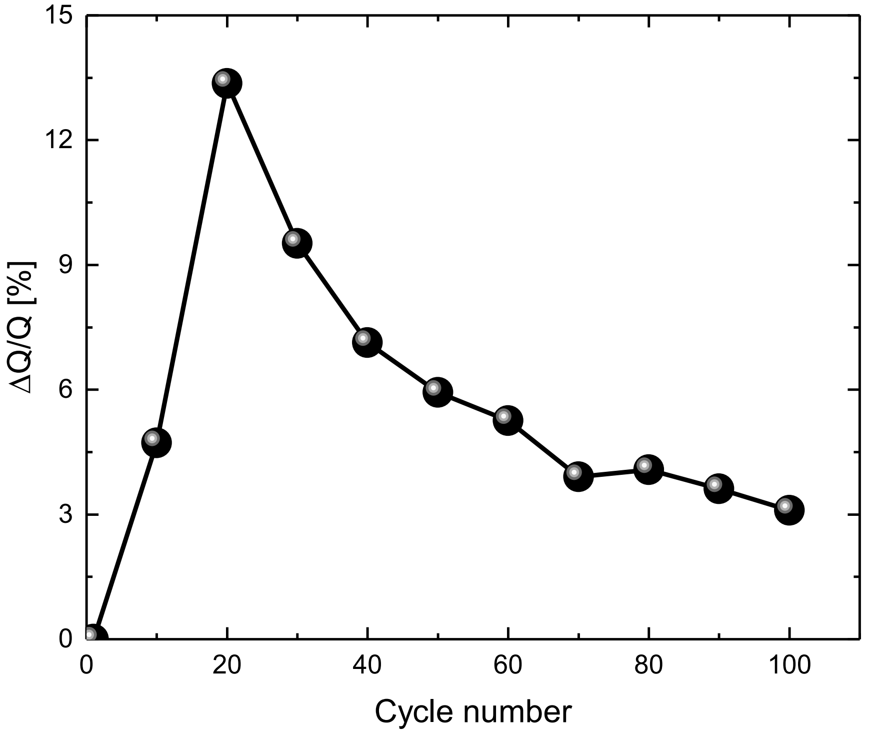

Figure S1. Growth rate of the area of the CV as function of the cycle number during the electrochemical oxidation of the Ni nanoparticles by 100 CV cycles at 50 mV s^{-1} in the range -0.2÷0.8 V in 1 M KOH solution.

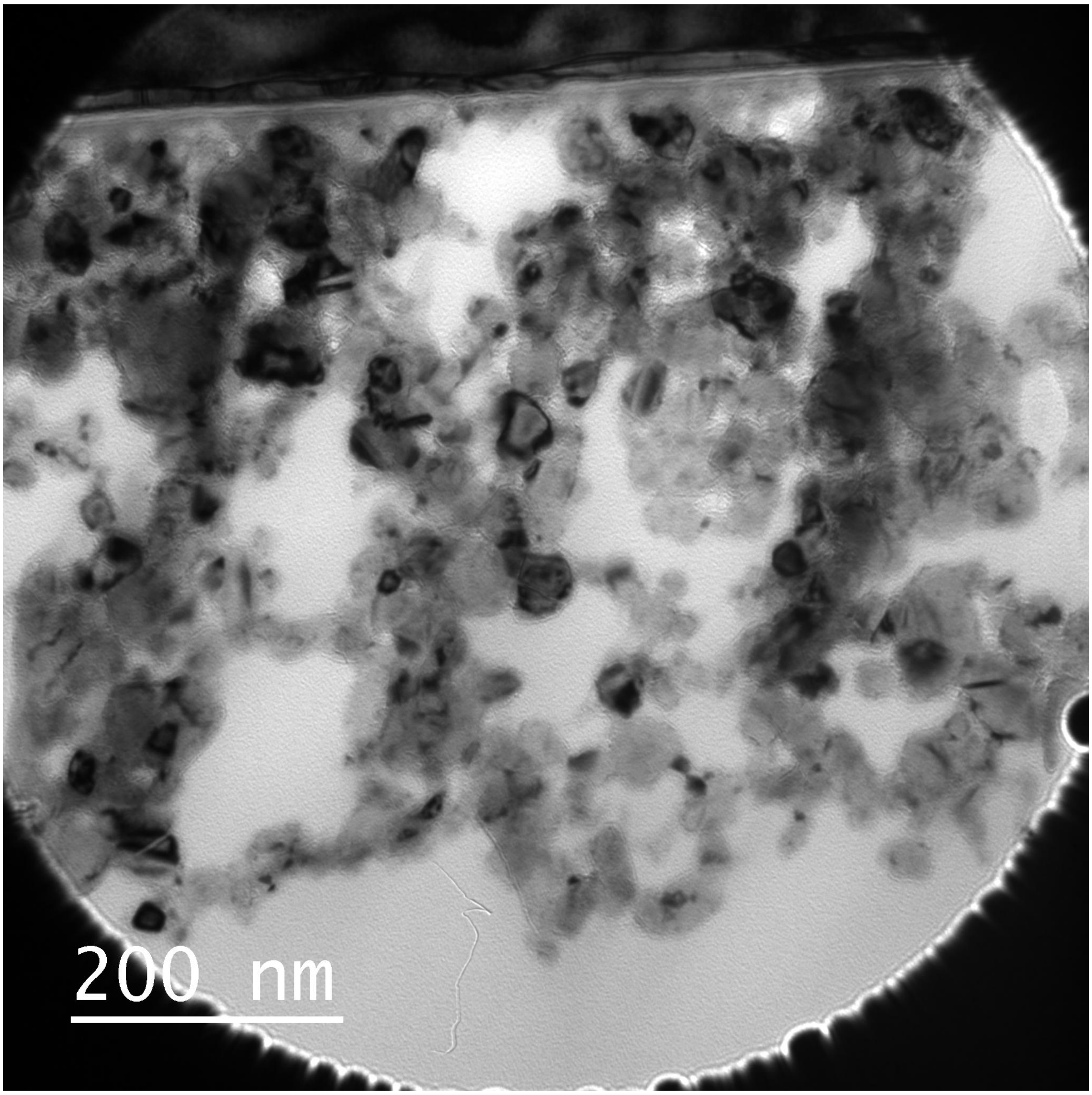

Figure S2. Low-magnification TEM image in bright field mode of the $Ni(OH)_2$@Ni core-shell nanochains.

In this work, accurate mass measurements were performed and the obtained results are reported in Table S1.

Sample	Mass [mg cm^{-2}]	#Ni [10^{18} atoms cm^{-2}]	% Ni
$Ni(OH)_2$ nanowalls	1.68 ± 0.04	10.9	/
Ni nanoparticles	0.85 ± 0.01	8.7	100
$Ni(OH)_2$@Ni core-shell nanochains	1.02 ± 0.04	/	/
$Ni(OH)_2$ shell (experimental)	0.43 ± 0.01	2.8	32
$Ni(OH)_2$ shell (estimated)	0.47 ± 0.05	3.1	36
Ni core (estimated)	0.55 ± 0.09	5.6	64

Table S1. $Ni(OH)_2$ mass measurements.

$Ni(OH)_2$ nanowalls mass was measured as mass difference of samples before and after CBD. Ni nanoparticles mass was measured as mass difference of samples before CBD and after thermal annealing.

Once assumed that the mass difference between $Ni(OH)_2$@Ni core-shell nanochains and Ni nanoparticles (Δm [g cm^{-2}]) is due to OH^- incorporation, $Ni(OH)_2$ shell mass can be estimated by the following equation

$$m_{Ni(OH)_2\ shell\ (estimated)} = \frac{\Delta m}{2\ M_{OH^-}} M_{Ni(OH)_2}$$

where M_{OH^-} and $M_{Ni(OH)_2}$ are the molar mass of OH^- (17.0 g mol^{-1}) and $Ni(OH)_2$ (92.7 g mol^{-1}) respectively. A value of 0.47 ± 0.05 mg cm^{-2} was found.

$Ni(OH)_2$ shell mass was also measured experimentally following the procedure proposed by Dai et al. to improve result accuracy [1]. A $Ni(OH)_2$@Ni core-shell nanochains was immersed in a 10 M NH_3 solution for 24 hours at room temperature. Since ammonia molecules coordinate Ni^{2+} ions, forming the nickel ammonia complex, $Ni(OH)_2$ dissolved [2]:

$$Ni(OH)_2 + 6NH_3 \rightarrow [Ni(NH_3)_6]^{2+} + 2OH^-.$$

$Ni(OH)_2$ shell mass was further evaluated as the mass difference before and after the immersion in the ammonia solution. A value of 0.434 mg cm^{-2} was obtained, which is consistent with the expected one. In this way a possible contribution of the Ni foam substrate is also considered, leading to more reliable results.

Ni areal density [10^{18} atoms cm^{-2}] was calculated for the different samples and reported in Table S1.

CV was performed at different scan rates (1-50 mV s^{-1}) in the potential range -0.2÷0.8 V in 1 M KOH. Figure S2 reports the recorded CV curves.

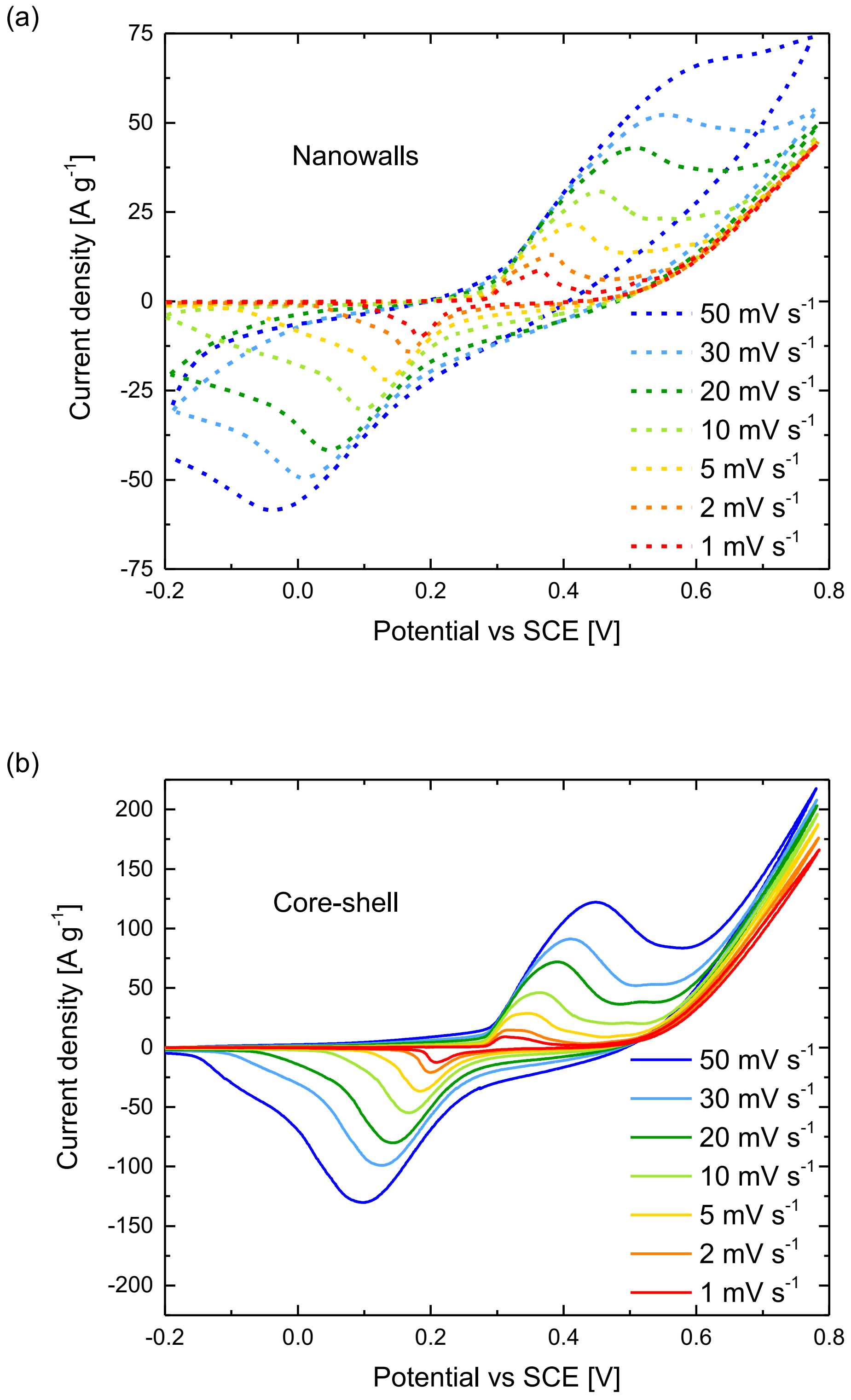

Figure S3. CV curves of nanowalls (a) and core-shell (b) at different scan rates in the potential range -0.2÷0.8 V in 1 M KOH.

GCD measurements were performed at different current densities (1-64 A g^{-1}) in 1 M KOH. The corresponding charge and discharge profiles are shown in Figure S4.

(a)

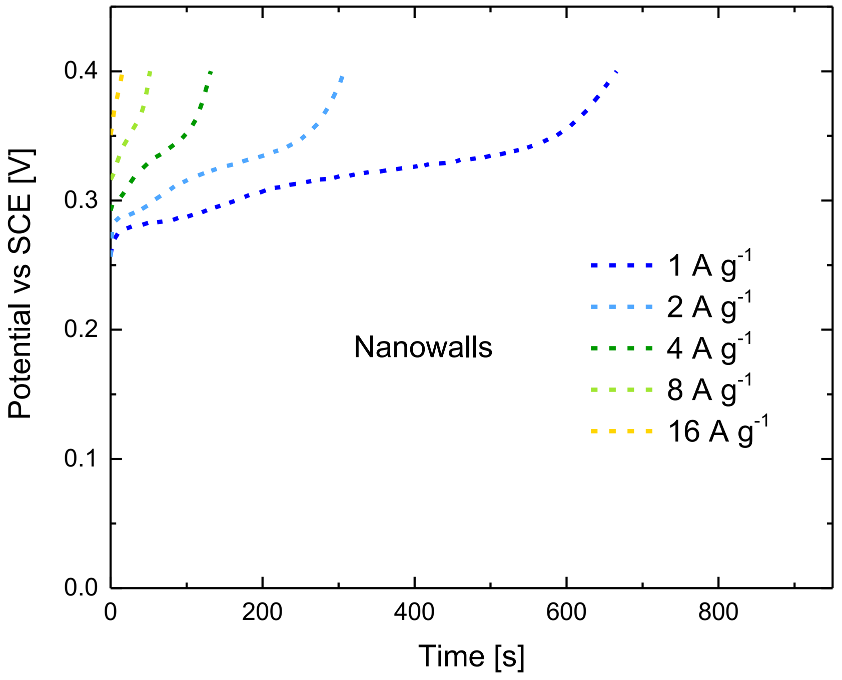

(b)

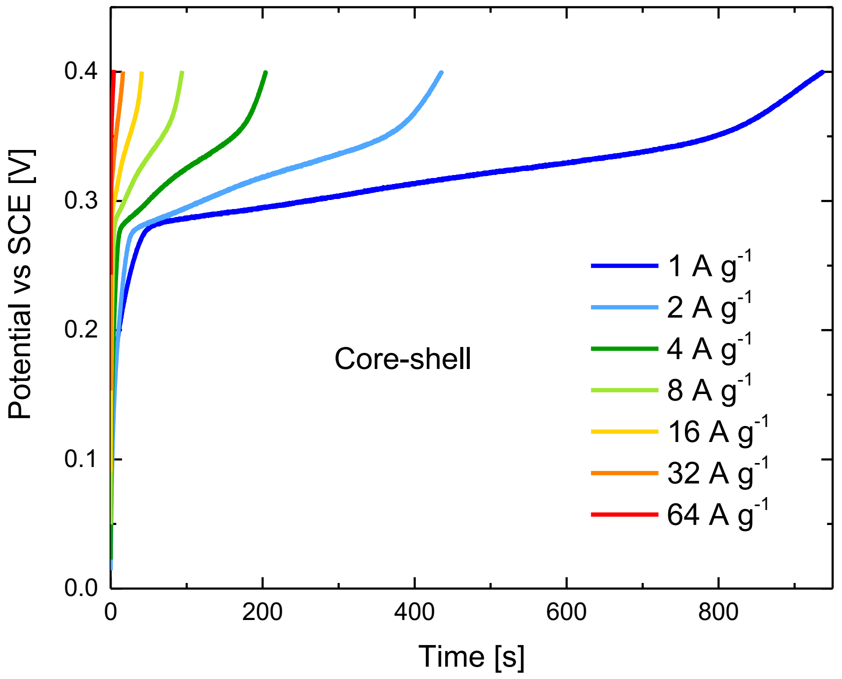

(c)

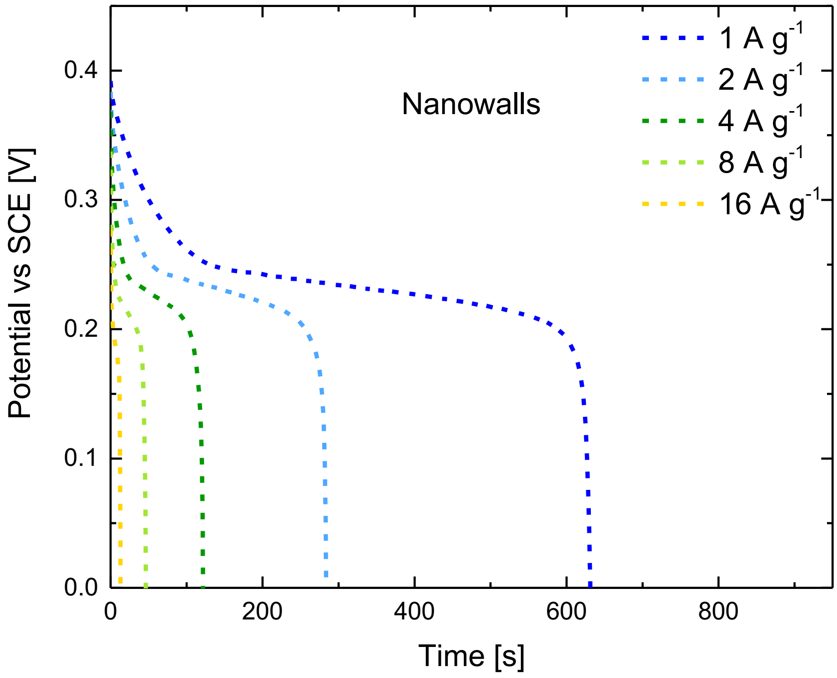

(d)

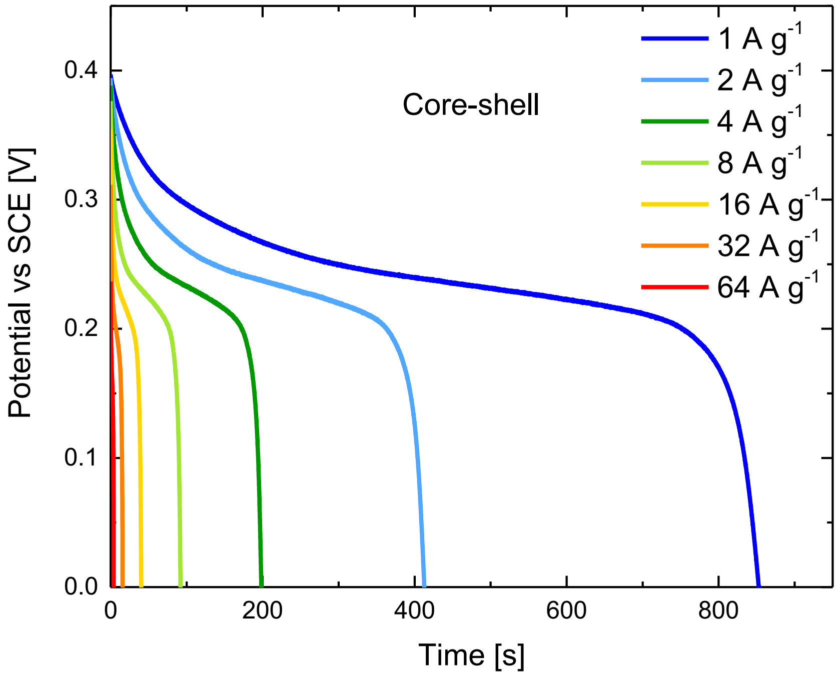

Figure S4. Charge (a) and discharge (c) curves of nanowalls, charge (b) and discharge (d) of core-shell measured at different current densities in 1 M KOH solution.

Specific capacity Q_s [mAh g^{-1}] was calculated form reduction (discharge) peak in CV curves according to the following equation [3]

$$Q_s = \frac{\int I\, dV}{m\, \frac{dv}{dt}}$$

where I is the current density [A cm^{-2}], m is the mass of the active material [g cm^{-2}], and $\frac{dv}{dt}$ is the scan rate [V s^{-1}]. Figure S4 shows the specific capacitance as function of the scan rate for the nanowalls and core-shell electrodes.

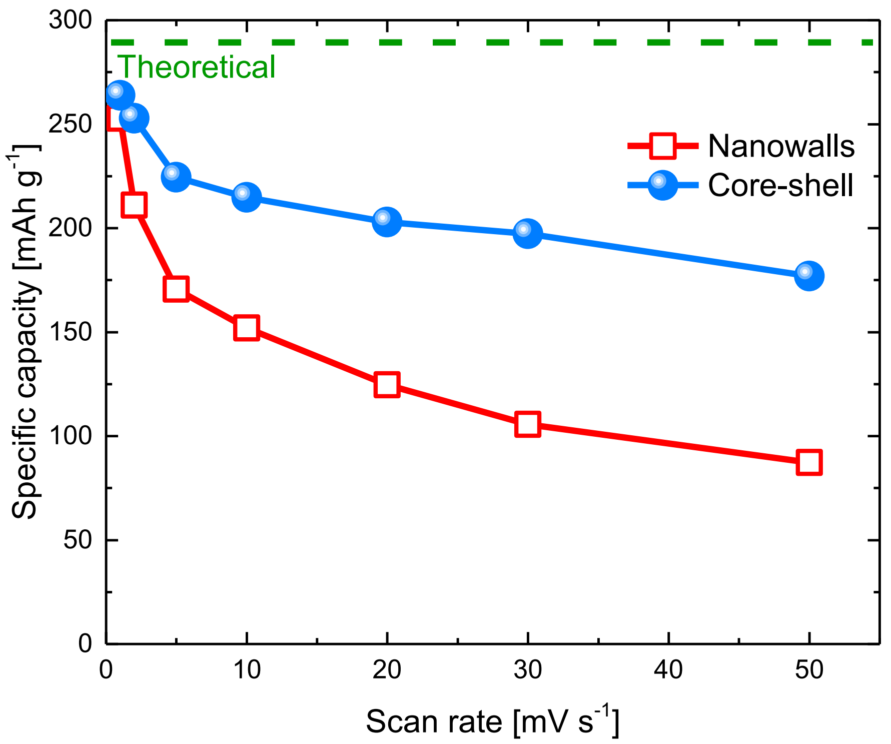

Figure S5. Specific capacity of nanowalls (red open squares) and core-shell (blue spheres) as function of scan rates.

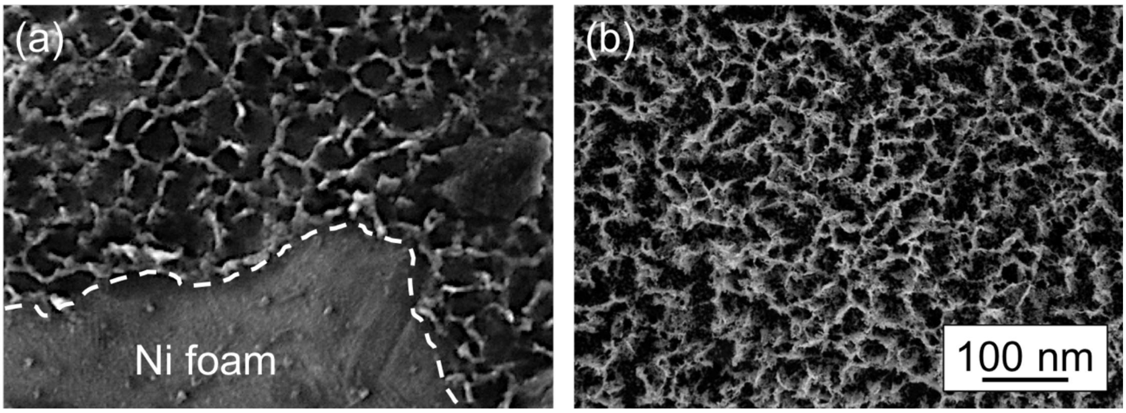

Figure S6. SEM images of (a) $Ni(OH)_2$ nanowalls and (b) $Ni(OH)_2$@Ni core-shell nanochains after cycling tests.

To further investigate the electrical properties of samples, we simulated the electric field in nanowalls and core-shell electrodes by using the COMSOL Multiphysics software. To simulate the nanowalls electrode, a 20 nm thick $Ni(OH)_2$ nanosheet was designed, while a 17 nm thick Ni nanosheet surrounded by a 3 nm thick $Ni(OH)_2$ shell was designed to simulate the core-shell electrode. A thick Ni substrate was used as current collector for both electrodes. The following room temperature conductivities were used: 10^{-13} S cm^{-1} for $Ni(OH)_2$, 10^5 S cm^{-1} for Ni, and 0.2 S cm^{-1} for 1 M KOH [28,30]. A steady-state model was employed to evaluate the electric field in the electrodes at a constant applied voltage (0.4 V). The simulation software used three different equations. In a stationary coordinate system, a slightly more general form of Ohm's law states that

$$J = \sigma E + J_e$$

where J is the current density, σ is the electrical conductivity, E is the electric field and J_e is the externally generated current density. Under static conditions, the electric field E is finally defined as

$$E = -\nabla V$$

where V is the electric potential. To handle current sources, the first equation can be generalized to

$$\nabla \cdot J = -\nabla \cdot (\sigma \nabla V - J_e) = Q_j$$

The diffusion coefficient of the OH^- ions for the $Ni(OH)_2@Ni$ core-shell nanochains was calculated as follows: i) anodic and cathodic peak current was plotted as function of the square root of the scan rate (Figure S5) ii) linear fit was performed; iii) the diffusion coefficient was calculated from the slope of the linear fit based on the Randles-Sevcik equation [4]

$$i_p = 0.4463\, nFAC \left(\frac{nFvD}{RT}\right)^2$$

where i_p is the peak current [A], n is the number of electrons involved in the redox event, F is the Faraday constant (96485 C mol^{-1}), A is the area of the electrode [cm^2], C is the concentration of the diffusing specie [mol cm^{-3}], v is the scan rate [V s^{-1}], D is the diffusion coefficient [cm^2 s^{-1}], R is the gas constant (8.314 J mol^{-1} K^{-1}) and T is the temperature. The OH^- ions diffusion coefficient was calculated for both anodic and cathodic peaks, finding an average value of $(1.415 \pm 0.002) \times 10^{-6}$ cm^2 s^{-1}.

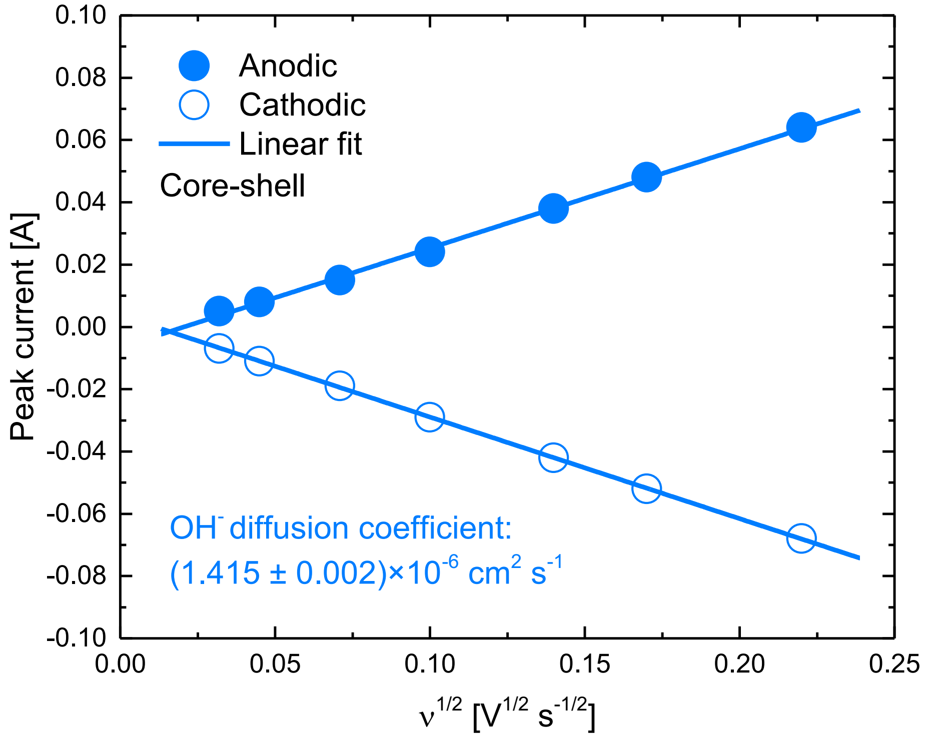

Figure S7. Anodic (spheres) and cathodic (open circles) peaks current of core-shell as function of scan rates.

References

[1] Dai, X. et al. $Ni(OH)_2/NiO/Ni$ composite nanotube arrays for high-performance supercapacitors. *Electrochim. Acta* **154,** 128-135 (2015).

[2] Van Bomme, A.; Dahn, J. R. Analysis of the Growth Mechanism of Coprecipitated Spherical and Dense Nickel, Manganese, and Cobalt-Containing Hydroxides in the Presence of Aqueous Ammonia. *Chem. Mater.* **21,** 1500–1503 (2009).

[3] Alhebshi, N. A.; Rakhi, R. B.; Alshareef, H. N. Conformal coating of $Ni(OH)_2$ nanoflakes on carbon fibers by chemical bath deposition for efficient supercapacitor electrodes. *J. Mater. Chem. A* **1,** 14897–14903 (2013).

[4] Bard, A. J., Faulkner, L. R., John Wiley & Sons, New York, 2001.
